# Role of (myo)fibroblasts in the development of vascular and connective tissue structure of the C38 colorectal cancer in mice

**DOI:** 10.1186/s40880-018-0316-x

**Published:** 2018-07-05

**Authors:** Edina Bugyik, Vanessza Szabó, Katalin Dezső, András Rókusz, Armanda Szücs, Péter Nagy, József Tóvári, Viktória László, Balázs Döme, Sándor Paku

**Affiliations:** 1 First Department of Pathology and Experimental Cancer Research, Semmelweis University, Budapest, Üllői út 26, 1085 Hungary; 2 Department of Experimental Pharmacology, National Institute of Oncology, Budapest, 1122 Hungary; 3 Department of Thoracic Surgery, Semmelweis University-National Institute of Oncology, Budapest, 1122 Hungary; 4 Department of Thoracic Surgery, Medical University of Vienna, Waehringer Guertel 18-20, 1090 Vienna, Austria; 5 Department of Biomedical Imaging and Image-guided Therapy, Medical University of Vienna, 1090 Vienna, Austria; 6 National Koranyi Institute of Pulmonology, Budapest, 1122 Hungary; 7 Tumor Progression Research Group, Hungarian Academy of Sciences-Semmelweis University, Budapest, 1085 Hungary

**Keywords:** Metastasis, Vasculature, Myofibroblasts, Incorporation

## Abstract

**Background:**

It remains unclear if the vascular and connective tissue structures of primary and metastatic tumors are intrinsically determined or whether these characteristics are defined by the host tissue. Therefore we examined the microanatomical steps of vasculature and connective tissue development of C38 colon carcinoma in different tissues.

**Methods:**

Tumors produced in mice at five different locations (the cecal wall, skin, liver, lung, and brain) were analyzed using fluorescent immunohistochemistry, electron microscopy and quantitative real-time polymerase chain reaction.

**Results:**

We found that in the cecal wall, skin, liver, and lung, resident fibroblasts differentiate into collagenous matrix-producing myofibroblasts at the tumor periphery. These activated fibroblasts together with the produced matrix were incorporated by the tumor. The connective tissue development culminated in the appearance of intratumoral tissue columns (centrally located single microvessels embedded in connective tissue and smooth muscle actin-expressing myofibroblasts surrounded by basement membrane). Conversely, in the brain (which lacks fibroblasts), C38 metastases only induced the development of vascularized desmoplastic tissue columns when the growing tumor reached the fibroblast-containing meninges.

**Conclusions:**

Our data suggest that the desmoplastic host tissue response is induced by tumor-derived fibrogenic molecules acting on host tissue fibroblasts. We concluded that not only the host tissue characteristics but also the tumor-derived fibrogenic signals determine the vascular and connective tissue structure of tumors.

## Introduction

The mechanism of vascularization in primary and metastatic tumors has long been debated. The tumor vascular pattern has been proposed to be indicative of the histologic type of the tumor [1]. Konerding et al. [2] examined the vascular structure of subcutaneous tumors using four tumor cell lines of different origins. They found that the vascular structure is characteristic of the individual tumor and showed that the vascular structure does not depend on the size and/or the rate of lesion growth but rather on the tumor type. In contrast, Solesvik et al. [3] investigated human malignant melanoma xenografts, and despite the identical histological type, different vascular patterns were found. They also observed that slow-growing melanomas had higher necrotic fractions and lower vessel volume per intact tumor volume than rapidly growing tumors.

In addition to tumor type, the extracellular matrix (collagen and basement membrane) structure of the host tissue can also have an influence on the vascular and connective tissue structure of the tumor. It was demonstrated that in brain metastases, the capillary basement membrane (BM) is the primary substrate for adhesion, migration and growth of the extravasated cells [4–6]. It was shown that the highly metastatic Lewis lung carcinoma (3LL-HH) tumor cell line uses the cellular side of the BM as a substrate for spreading during invasion of muscle, peripheral nerve and adipose tissue [7]. During this process, host cells are detached from their BM and become degraded; however, their BM remains intact. Tumor cell migration on the cellular side of the BM also plays an important role in the vascularization of 3LL-HH liver metastases [8].

In liver [9, 10], brain [11] and lung [12] metastases, it was shown that the differentiation grade of the tumor can also have an impact on the histologic structure of the metastases. Three different growth patterns were described in liver metastases of colorectal adenocarcinomas [9]. In the replacement growth pattern (high grade), the structure of the liver is preserved. However, in desmoplastic and pushing growth patterns, the structure of the liver is disturbed. In the pushing growth pattern, liver plates are pushed aside. As a result, compressed liver parenchyma surrounds the metastases. In the desmoplastic growth, a robust fibrous capsule separates the liver parenchyma from the tumor tissue.

Earlier, we described the development of the vasculature in a “pushing-type” experimental colorectal carcinoma model (C38) in the liver [13]. During the growth of metastases, smooth muscle actin (SMA)-positive cells appeared at the tumor-parenchyma interface, while hepatocytes disappeared from this region. This process resulted in the appearance of vascular lakes formed by the fusion of hepatic sinusoids at the border of the metastases. Fused sinusoids and collagenous matrix-producing SMA-positive myofibroblasts became incorporated into the growing tumor. The deepest part of the invagination was separated from the surrounding host tissue, and the process culminated in the formation of connective tissue columns with a centrally located, functional vessel. We believe that the formation of these columns is a characteristic feature of the C38 tumor. However, in experimental brain metastases, these structures were not present. Instead, the brain microvessels were directly surrounded by the tumor cells. This raises the question of whether the appearance of vessel-containing columns is a consequence of the host tissue’s microenvironmental effects on the tumor or, alternatively, whether these structures are intrinsic characteristic features of certain tumor types. Thus, their presence is independent from the target host tissue microenvironment. The main goal of the present study was to clarify whether the vascular and connective tissue structure of tumors are intrinsically determined by the tumor type or, alternatively, if these features are defined by the host tissue. We generated experimental tumors (C38 colon cancer) at five different locations (skin, cecal wall, liver, brain and lung) and analyzed their vascular and connective tissue structures. As the formation and remodeling of myofibroblast-containing connective tissue columns require active fibrogenesis, we also investigated the possible role of fibrogenic growth factors during column formation.

## Materials and methods

### Animals and tumor cell line

Eight-week-old male C57Bl/6 mice were obtained from the animal facility of the First Department of Pathology and Experimental Cancer Research of Semmelweis University (Budapest, Hungary). The animal study protocols were conducted according to National Institute of Health (NIH) guidelines for animal care and were approved by the Animal Care and Use Committee of Semmelweis University (PEI/001/2457-6/2015). The C38 colorectal carcinoma cell line was maintained in vivo by serial subcutaneous transplantations, as described previously [13]. Subcutaneous tumors were removed and cut into small pieces (~ 0.5 cm^3^) and were implanted under the skin of C57Bl/6 mice.

### In vivo experiments

To generate experimental tumors (subcutaneous tissue, cecal wall, brain, liver, and lung), subcutaneously growing C38 tumor tissue was removed, cut into small pieces (~ 2 mm^3^), and digested in RPMI-1640 medium (Cat. No.: R8758, Sigma-Aldrich, St Louis, MO, USA) supplemented with 0.7 mg/mL collagenase (Cat. No.: C5138, Sigma-Aldrich) at 37 °C, for 45 min. After filtration through fourfold sterile gauze, cells were centrifuged (800 rpm, 10 min, 4 °C). The pellet was resuspended in 10 mL of RPMI-1640 medium without any supplement, and the number of viable tumor cells was counted using the trypan blue exclusion test. Mice were anesthetized with an intraperitoneal injection of ketamine–xylazine (Cat. No.: K113, 80:12 mg/kg; Sigma-Aldrich).

In the orthotopic primary tumor model, a midline incision was made in the abdomen, and the cecum was gently exteriorized onto gauze impregnated with saline. Cells were injected into the cecal wall of mice with a 30-gauge needle (Braun, Melsungen, Germany) in ~ 5 µL volume (~ 2 × 10^4^ cells). The cecum was returned to the abdominal cavity, and the incision was closed.

Cells were injected heterotopically into the brain, spleen, and footpad of the mice. Brain tumors were produced as described previously [11]. Briefly, the right parietal bone was drilled with a 21-gauge needle (Braun) 2 mm posterior to the coronal suture and 1 mm lateral to the sagittal suture. Ten thousand cells in a volume of 2 µL were slowly injected using a 10 µL Hamilton syringe. Liver metastases were produced by injecting tumor cells (2 × 10^5^ cells in a volume of 50 µL) into the spleen of mice as described previously [13]. To produce lung metastases, cell suspension (5 × 10^4^ cells in a volume of 20 µL) was injected into the footpads of the hind legs of the mice. The legs were amputated 18–28 days following tumor cell injection.

Subcutaneous tumors were generated by implanting 0.5 cm^3^ tumor pieces under the skin of the mice.

Animals were sacrificed 7–10 days after intracranial injection, 15–18 days after intrasplenic injection, 15–21 days after subcutaneous transplantation, 21 days after orthotopic injection, and 5–8 weeks after hind leg amputation.

### Immunofluorescence analysis

Tumors from the five different locations were removed and frozen in isopentane (Sigma-Aldrich) chilled with liquid nitrogen. Frozen sections (15 µm) were fixed in methanol (− 20 °C) for 10 min and incubated at room temperature (1 h) with a mixture of primary antibodies (Table 1). After washing, sections were incubated (30 min) with appropriate secondary antibodies (Life Technologies, Carlsbad, CA, USA) (Table 1). Samples were analyzed by confocal laser scanning microscopy using a Bio-Rad MRC-1024 system (Bio-Rad, Richmond, CA, USA).

**Table 1:** Antibodies and fluorescent dye used for immunofluorescence

Antibody	Species	Manufacturer	Catalog number	Dilution
CD31	Rat monoclonal	BD Pharmingen, Franklin Lakes, NJ, USA	550275	1:50
Laminin	Rabbit polyclonal	DAKO, Glostrup, Denmark	Z0097	1:200
Collagen I	Rabbit polyclonal	Chemicon, Billerica, MA, USA	AB765P	1:100
SMA	Mouse monoclonal	DAKO, Glostrup, Denmark	M0851	1:200
panCK-FITC	Mouse monoclonal	DAKO, Glostrup, Denmark	F0859	1:100
TOTO-3		Invitrogen, Carlsbad, CA, USA	T3604	1:500

### Morphometric analysis

Frozen sections of tumor samples from all locations were stained for CD31 and laminin. Sections were scanned using Pannoramic Scanner (3D-Histech Ltd., Budapest, Hungary), and a morphometric analysis was performed using Pannoramic Viewer software (3D-Histech Ltd.). Only the columns containing one individual vessel were used during measurements. The distance between the basement membrane (BM) of the central vessel and the laminin deposited by the tumor cells around the column was measured at two sides of the vessel. The orientation of the columns in the tumor tissues was random, resulting in cut profiles of different ovality. Therefore, we always determined the smallest distance between the central vessel and the column edge. At least 5 mice/tumor location and three slides from each tumor were used. Ten to twenty vessels/slide were measured.

### Electron microscopy

Tumor-bearing mice were anesthetized and perfused via the left ventricle with 1× phosphate-buffered saline (PBS) for 10 min and with a mixture of 4% paraformaldehyde and 1% glutaraldehyde in PBS (pH 7.2) for 15 min at room temperature. Tissues containing tumors (cecal wall, brain, liver, lung, and subcutaneous tissue) were removed, cut into 1–2 mm pieces, and immersed in the same fixative for an additional 2 h. Pieces were postfixed in 1% OsO_4_ and 0.5% K-ferrocyanide in PBS for 2 h, dehydrated in a graded series of acetone, and embedded in Spurr’s mixture. Semi-thin sections of samples stained by 0.5% toluidine blue (pH 8.5) were analyzed. Ultrathin sections cut by an RMC MT-7 ultramicrotome (Research and Manufacturing Co, Tucson, AZ) were contrasted with uranyl-acetate and lead citrate and analyzed using a Philips CM10 electron microscope (Philips, Eindhoven, The Netherlands).

### Cell lines

B16 mouse melanoma cells were cultured in RPMI-1640 supplemented with 10% fetal bovine serum (Sigma-Aldrich), and 1 × 10^6^ tumor cells were collected in lysis buffer.

To obtain a cell culture from the dissociated C38 tumor, 2.5 × 10^5^ viable tumor cells were seeded into T-25 flasks containing 5 ml of complete media and incubated at 37 °C in 5% CO_2_. The cells were subcultured every 3 days by a conventional trypsinization method, and 1 × 10^6^ tumor cells were collected in lysis buffer.

### Gene expression analysis

Total RNA was isolated with Trizol (Cat. No.: 15596–018, Life Technologies, Waltham, MA, USA). RNA concentration was measured by a NanoDrop 1000 Spectrophotometer (Thermo Fisher Scientific, Wilmington, DE), and 1 µg RNA per sample was converted into cDNA.

A high capacity cDNA reverse transcription kit (Cat. No.: 4368814, Thermo Fisher Scientific) was used for cDNA synthesis as recommended by the supplier. Quantitative real-time polymerase chain reaction. (qRT-PCR) was performed by the ABI Quant Studio3 (Thermo Fisher Scientific) sequence detection system using Thermo Fisher Scientific TaqMan gene expression assays (connective tissue growth factor (Ctgf): Mm01192931_g1; Fibroblast growth factor 2 (Fgf2): Mm00433287_m1; Transforming growth factor-β 1 (Tgfb1): Mm01178820_m1; Transforming growth factor-β2 (Tgfb2): Mm00436955_m1; Transforming growth factor-β3 (Tgfb3): Mm00436960_m1; Platelet-derived growth factor-β Pdgfb: Mm01298578_m1) according to the manufacturer’s instructions. Glyceraldehyde-3-phosphate dehydrogenase (GAPDH, Thermo Fischer Scientific, Cat No.: 4352932E) was used as an endogenous control. All samples were run in triplicate in a 20 µL reaction volume. The results were obtained as threshold cycle (CT) values. Expression levels were calculated using the ΔCT method. The values were calculated as the mean values of three independent measurements, and the expression levels of mRNA in all samples were defined as ratios to GAPDH expression (%).

### Statistical analysis

Data are represented as the mean ± SD of at least three independent experiments. The statistical significance of differences between groups was analyzed with Student’s *t* test. Values of *P* < 0.05 were considered statistically significant (GraphPad Software, La Jolla, CA).

## Results

### Incorporation of vessels and connective tissue at the tumor surface

**Fig. 1:**
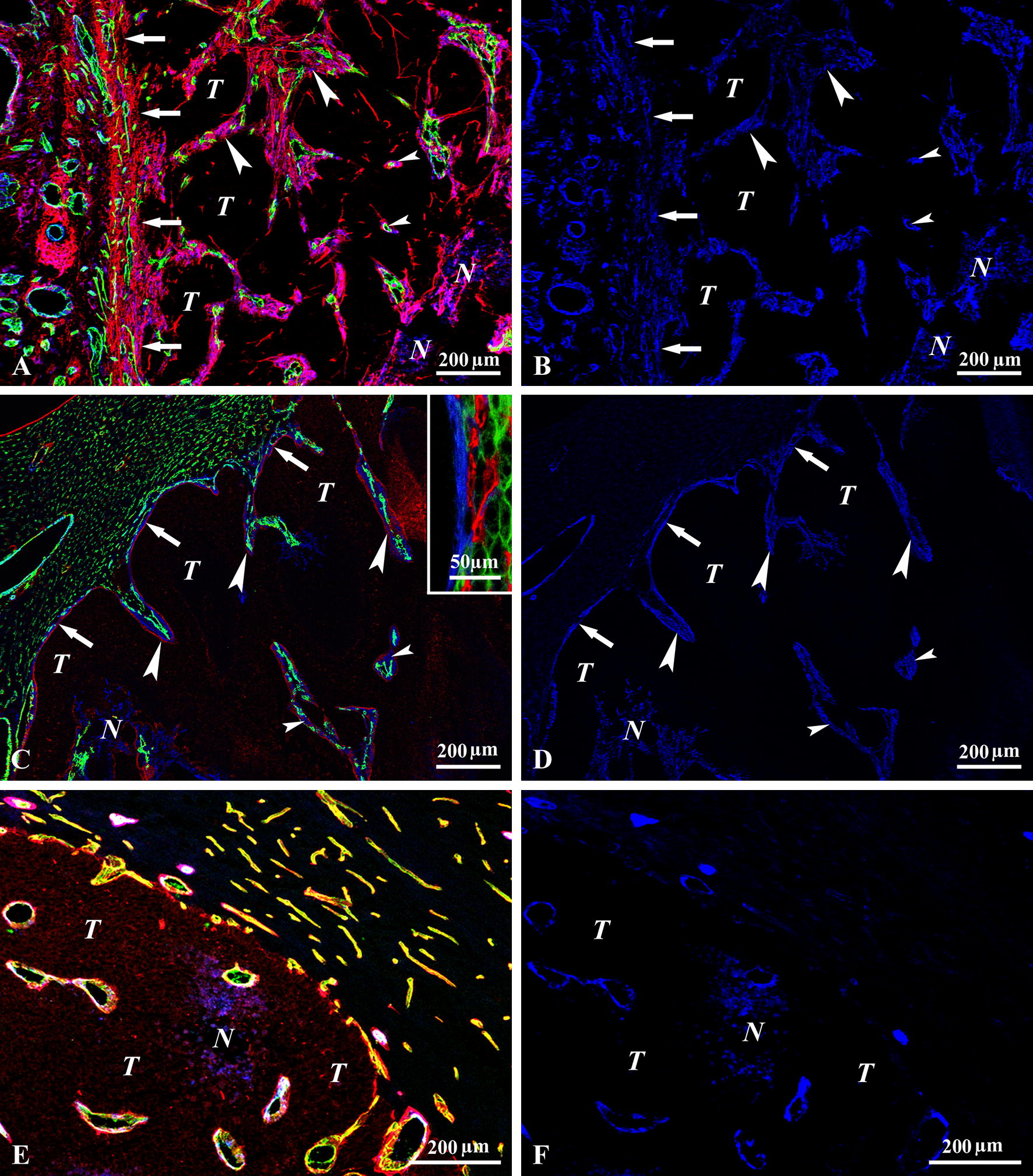
Accumulation and incorporation of vessels and connective tissue at the tumor surface. **A**, **B** Subcutaneously growing C38 tumor. The accumulation of collagen I (*red*)- and smooth muscle actin (SMA)-expressing myofibroblasts (*blue*) is present at the tumor surface (*arrows*). Black areas represent viable tumor mass (*T*) beneath the tumor surface. For clarity, **B** shows the blue (SMA) channel separately. Numerous vessels (CD31, *green*) are being incorporated (together with connective tissue) by the tumor (*large arrowheads*). Smaller connective tissue columns separated from the incorporated connective tissue are visible within the tumor tissue (*small arrowheads*). *N* necrosis. **C**, **D** C38 tumor (*T*) growing in the liver. In the left upper part of **c**, the peritumoral liver parenchyma—with the dense CD31-positive network of sinusoids (green)—is visible. The tumor-parenchyma interface is indicated by arrows. Here, accumulation of SMA-positive cells can be observed (see also **D**, for the separate blue channel). These cells are also present in the invaginations (*large arrowheads*) and in the incorporated host connective tissue deep within the tumor (*small arrowheads*). The invaginations and the incorporated host tissue pieces are delineated by the laminin (*red*) deposited by the tumor cells. Inset: high-power micrograph of the surface of a C38 liver metastasis, demonstrating the accumulation of SMA-positive cells (*blue*). Tumor tissue is present on the left side (*black area*). On the right side, pan-cytokeratin-expressing hepatocytes (*green*) and CD31-positive vessels (*red*) are visible. Between the hepatocytes and the tumor tissue, SMA-positive cells (*blue*) can be observed. **E**, **F** A C38 brain metastasis (**E**) is highlighted and bordered by laminin (*red*), which is deposited by tumor cells. CD31-positive vessels (*green*) appear yellowish because of their close proximity of the basement membrane (*red*) (**E**). No SMA accumulation is present at the periphery of the tumor, as can be seen on the separate blue (SMA) channel (**F**). The peritumoral vessels are smaller than the intratumoral vessels, which are surrounded by SMA-positive pericytes (*blue*). SMA-positive cells are not found around the peritumoral vessels, excluding the arterioles. *N* necrosis, *T* tumor tissue

**Fig. 2:**
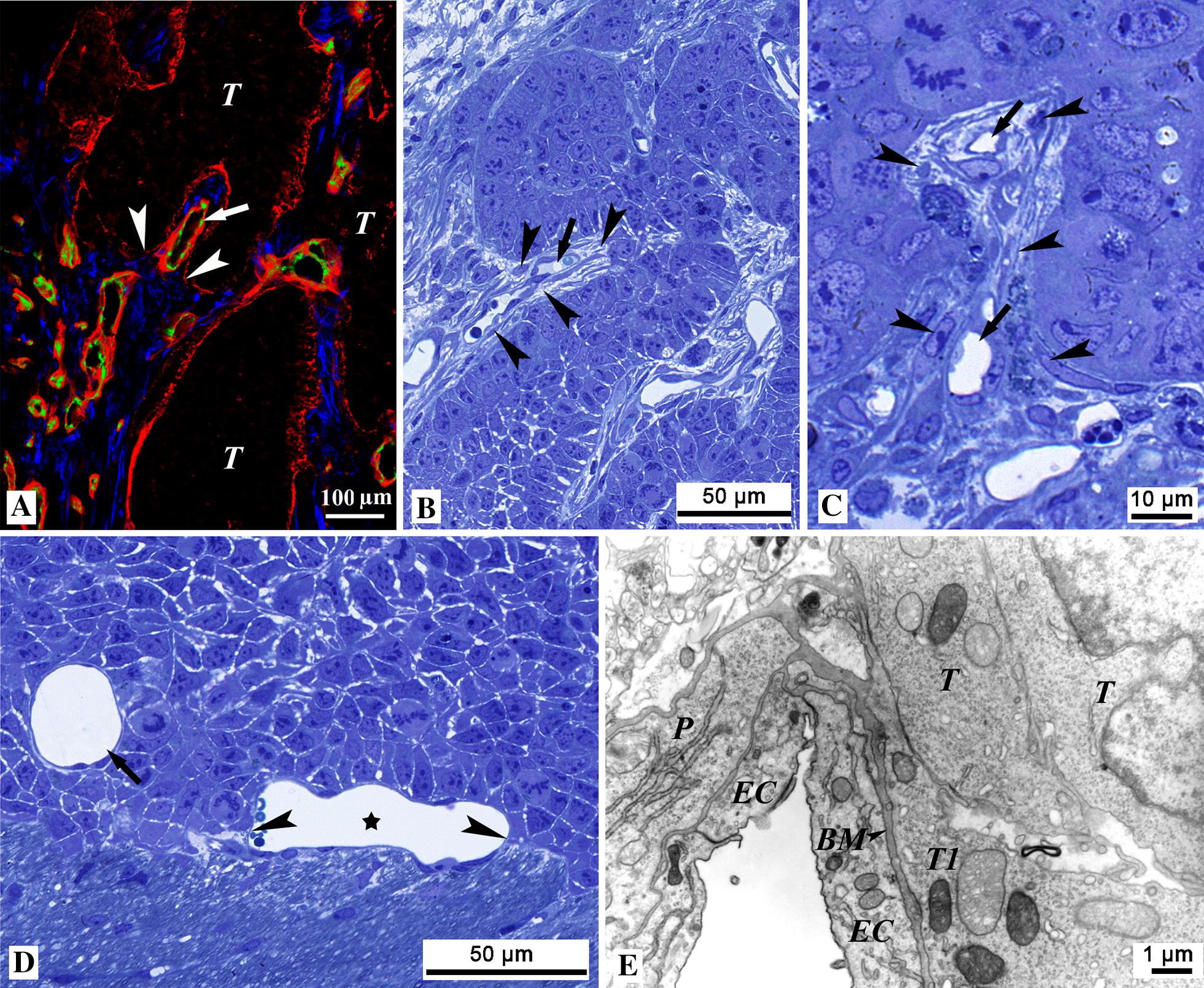
The process of vessel incorporation. **A** Frozen section of a subcutaneous C38 tumor stained for laminin (*red*), CD31 (*green*) and SMA (*blue*). The tumor tissue (*T*) appears black and is surrounded by laminin (*red*) deposited by the tumor cells. A large vessel (*arrow*) is being incorporated together with SMA-positive cells (*blue*) in an invagination. The outer part of the invagination, in contrast to the inner part, is delineated by a thinner and more fragmented basement membrane (*arrowheads*). **B**, **C** Semi-thin sections of C38 tumors growing in the subcutaneous tissue (**B**) and in the cecal wall (**C**). Blood vessels (*arrows*) and the surrounding connective tissue with cellular elements (*arrowheads*) are being incorporated by the tumor. **D** Semi-thin section of a C38 brain metastasis. A vessel (*asterisk*) is partially engulfed by the tumor mass (*arrowheads*). During this process, the brain parenchyma (located in the lower part of the picture) is excluded from the tumor (located in the upper part of the picture). The tumor cells are in close vicinity to the wall of the fully incorporated vessel (*arrow*). **E** An electron micrograph of a partially incorporated brain capillary at the surface of the tumor (*T*). A tumor cell (*T1*) is in touch with the basement membrane (*BM*) of the capillary. Collagen fibers cannot be observed between the tumor cell (*T1*) and the capillary basement membrane. *EC* endothelial cell, *P* pericyte

At the periphery of C38 tumors growing in subcutaneous tissue, liver, colon, and lung, we found smooth muscle actin (SMA)-expressing activated fibroblasts (myofibroblasts) and the consequently accumulated collagen (Fig. 1A–D). However, there was no myofibroblast or collagen accumulation around tumors growing in the brain, as only the pericytes of the intratumoral vessels were SMA-positive (Fig. 1E, F). Accordingly, although C38 tumors acquired their vasculature by the incorporation of the peritumoral host tissue at all tumor sites, the incorporation process in the brain was different from the incorporation process in the skin, colon, liver, and lung. For the latter organs, where SMA-positive cells and collagen accumulated around the tumors, invaginations of different sizes were formed at the surface of the tumors containing vessels and perivascular connective tissue (Figs. 1A, C, 2A–C). BM deposited by the tumor delineated the invaginations (Fig. 2A).

**Fig. 3:**
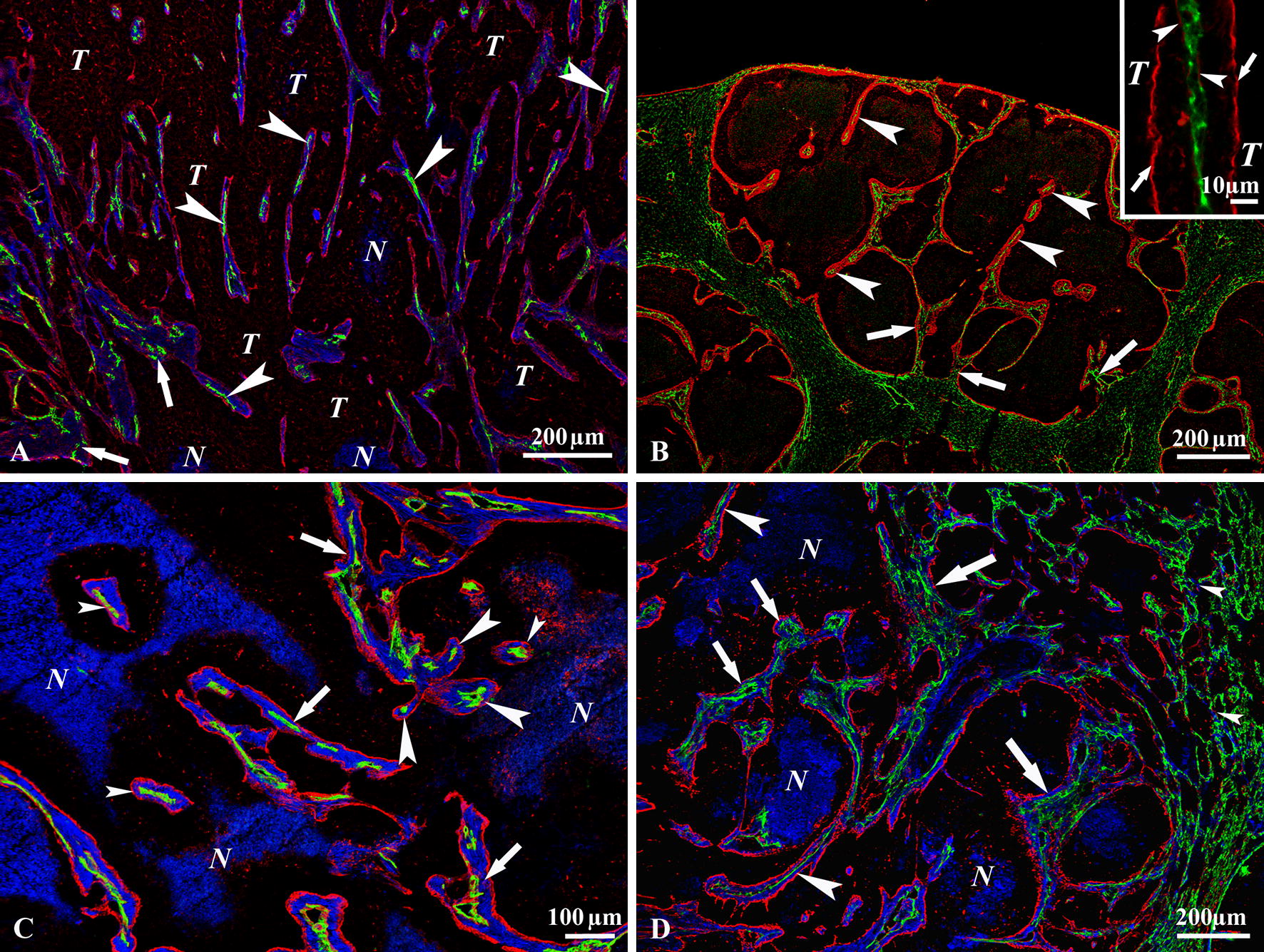
“Processing” of the invaginations. **A** A C38 tumor growing in the cecal wall. Incorporated connective tissue, which contains myofibroblasts (SMA, blue) and numerous vessels (CD31, green) (arrows) is visible throughout the tumor mass. All blue areas represent the columns, excluding necrotic areas (marked by N). Necrotic tissue is also highlighted by the nonspecific binding of the anti-mouse secondary antibody (used to detect the mouse monoclonal primary antibody against SMA) to these areas. Note the high number of advanced-stage connective tissue columns that contain only one single vessel (*arrowheads*). The columns are delineated by laminin (red) containing basement membrane produced by the tumor (T). **B** C38 metastasis in the liver. The early-stage incorporations contain numerous vessels (CD31, green) (*arrows*). Advanced-stage connective tissue columns, which contain single central vessels, are present intratumorally (*arrowheads*). Laminin (*red*) deposited by the tumor cells borders the incorporated tissue and the columns. The inset shows the fine structure of a longitudinally sectioned column containing a single central vessel (CD31, green). The basement membrane of the vessel is marked by small arrowheads. The basement membrane marked by small arrows is deposited by the tumor (T) at the surface of the column. **C** Subcutaneous C38 tumor. In addition to columns filled with myofibroblasts (SMA, *blue*) (*small arrowheads*) and containing a single central vessel (CD31, *green*), there are areas containing connective tissue columns with numerous vessels (*arrows*). Note the columns (*large arrowheads*) separated only partially from a larger connective tissue mass during the final steps of the maturation process. The incorporated connective tissue and the columns are bordered by laminin (*red*) deposited by the tumor cells. *N* marks areas of necrotic tissue that also appear blue, although this staining is due to the nonspecific binding of the anti-mouse secondary antibody used to detect the anti-SMA antibody. **D** C38 lung metastasis. The section is labeled with anti-CD31 (*green*), anti-laminin (*red*) and anti-SMA antibodies (*blue*). There are no SMA-positive cells (*blue*) in the alveolar walls of the normal lung tissue far from the metastasis (right edge of the micrograph). SMA-positive cells begin to appear in the peritumoral lung tissue (*small arrowheads*), but the number of these cells only notably increases intratumorally. Large early-stage connective tissue columns are present at the tumor periphery (*large arrows*), from which smaller tissue pieces containing different numbers of vessels are detaching (*small arrows*). *Large arrowheads* indicate mature columns with single vessels. *N* marks areas of necrotic tissue that also appear blue, although this staining is due to the nonspecific binding of the anti-mouse secondary antibody used to detect the anti-SMA antibody

The situation was slightly different in the lung tissue, where the tumor mass advanced through the alveoli, thereby incorporating the alveolar walls (with all of its components). This process resulted in invagination-like structures in the tumor (Fig. 3D).

In contrast, in tumors growing in the brain, the parenchyma (astrocytes) was completely separated from the vessels and excluded from the tumor (Fig. 2D). However, pericytes maintained their original position, and invaginations did not develop in the brain. Instead, the tumor tissue engulfed the cerebral capillaries one by one at the advancing margin. The tumor cells attached tightly to the capillary BM, and no deposited collagen could be observed between the vessels and the tumor cells (Fig. 2E).

### Maturation of the columns

**Fig. 4:**
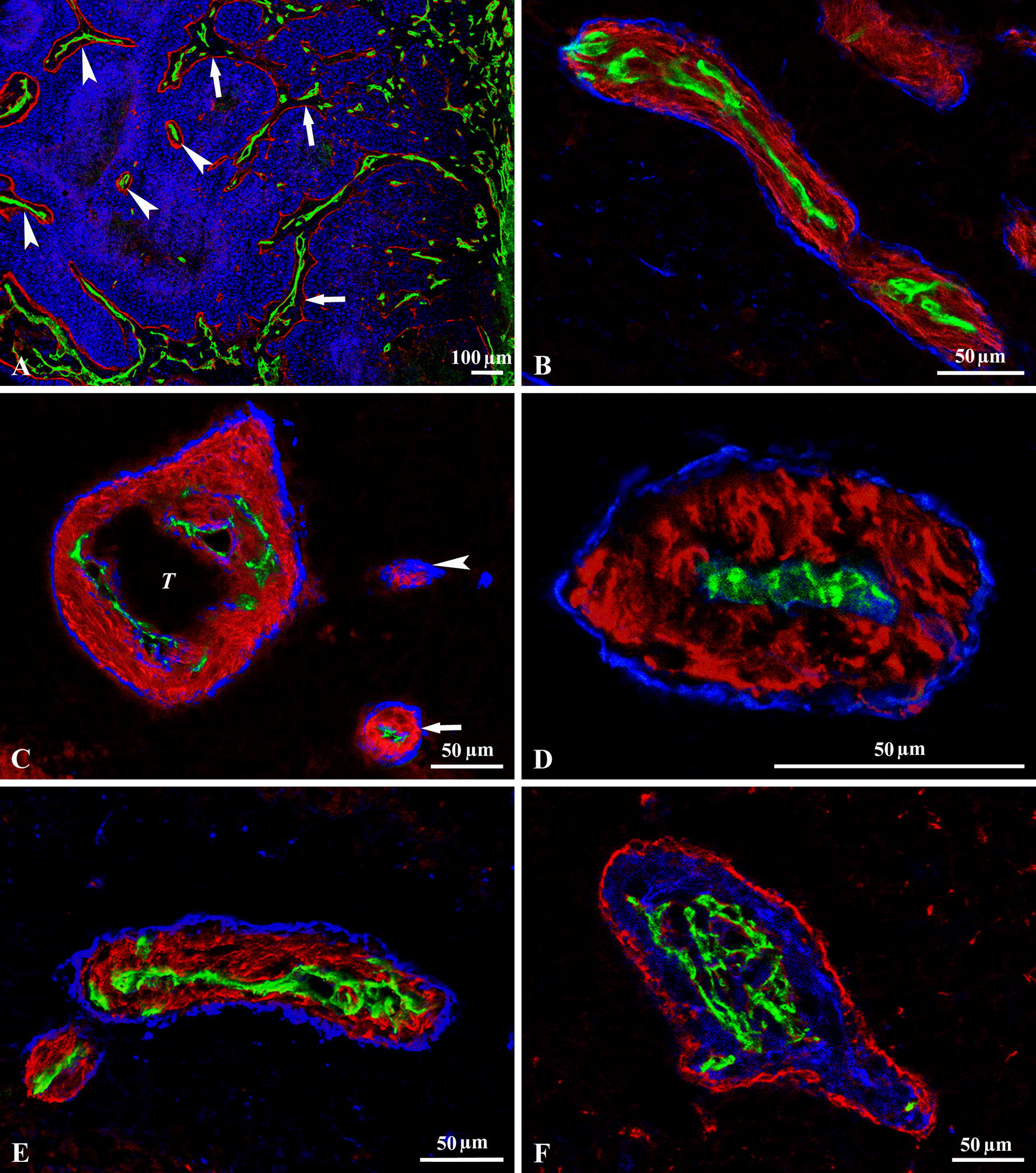
Maturation and final form of the columns. **A** Frozen section of a subcutaneous C38 tumor labeled for laminin (*red*), CD31 (*green*) and cell nuclei (TOTO-3, *blue*). The maturation process of the columns can be observed as we move from the peritumoral host tissue (right side of the picture) towards the center of the tumor (left side): the size of the columns and the amount of the deposited laminin increase from the periphery towards the tumor center. The newly incorporated connective tissue columns contain more vessels (*arrows*). After “processing” by tumor cells, they contain only a single vessel (*arrowheads*). **B** Tissue column in an orthotopically growing C38 tumor (black areas). The centrally located vessel (CD31, *green*) is surrounded by SMA-positive myofibroblasts (*red*) and enclosed by laminin (*blue*). **C** The frozen section of a C38 liver metastasis labeled with CD31 (*green*), SMA (*red*) and laminin (*blue*). Black areas represent tumor tissue. As maturation of the tissue columns progresses, the central part of the large column containing multiple vessels is invaded by the tumor (*T*). The tumor mass present within the column is not delineated by laminin, which indicates an early phase of invasion. At the lower right, a mature column is visible (*arrow*) containing a single vessel located centrally. Note the presence of a “misprocessed” column (*arrowhead*) that contains no vessels. **D** Connective tissue column in a subcutaneous C38 tumor (tumor cells fill the outer black area). The completely matured column contains a single central vessel (CD31, green) surrounded by connective tissue, which contains collagen I (red) and laminin deposited by the tumor cells (blue). **E** C38 lung metastasis. The black area represents tumor tissue. The column shows the same structure as in other locations. A central vessel can be seen (CD31, *green*), surrounded by SMA-positive cells (*red*) and laminin (*blue*). **F** C38 metastasis (black areas) reaching the brain surface. A connective tissue column that contains numerous vessels (CD31, *green*) surrounded by SMA-positive cells (*blue*) and laminin (*red*) produced by the tumor can be observed at this location

In early invaginations, the number of incorporated vessels and the amount of connective tissue were dependent on the size of the vessels and the invaginations (Figs. 1A, C, 3A–D, 4A). As invaginations with multiple capillaries moved deeper into the tumor tissue, tumor cells separated the microvessels from each other. This “maturation” process culminated in the appearance of connective tissue columns with a single central vessel (Figs. 3A–D, 4A–E). In detail, the cross-sectional view of the columns showed the following structural elements from inside out: endothelial layer, capillary BM, SMA-positive cells embedded in collagen-containing matrix, and BM of the tumor (Fig. 4B–F). We observed that both the size of the connective tissue columns and the amount of deposited perivascular BM material increased from the peritumoral host tissue towards the tumor center (Fig. 4A). We found the thickest connective tissue columns in subcutaneous tumors (18.9 ± 1.9 µm,) whereas the diameter of the columns at other locations (liver: 15.5 ± 1.7 µm*, lung: 12.2 ± 2.3 µm*, cecal wall: 13.2 ± 2.1 µm*) were significantly smaller (*t test, *P *≤ 0.05). The final consequence of the maturation process was that at all locations (where collagen and SMA-positive cells accumulated around the tumors), the same connective tissue and vascular structure developed.

As the brain parenchyma lacks fibroblasts, no connective tissue columns were produced in this location. However, we occasionally observed connective tissue columns also in the C38 cerebral metastases when the growing tumor reached the meningeal fibroblasts (Fig. 4F). However, we could only minimally detect columns that reached the single-vessel stage during their development.

### Relative gene expression analysis of fibrogenic growth factors

**Fig. 5:**
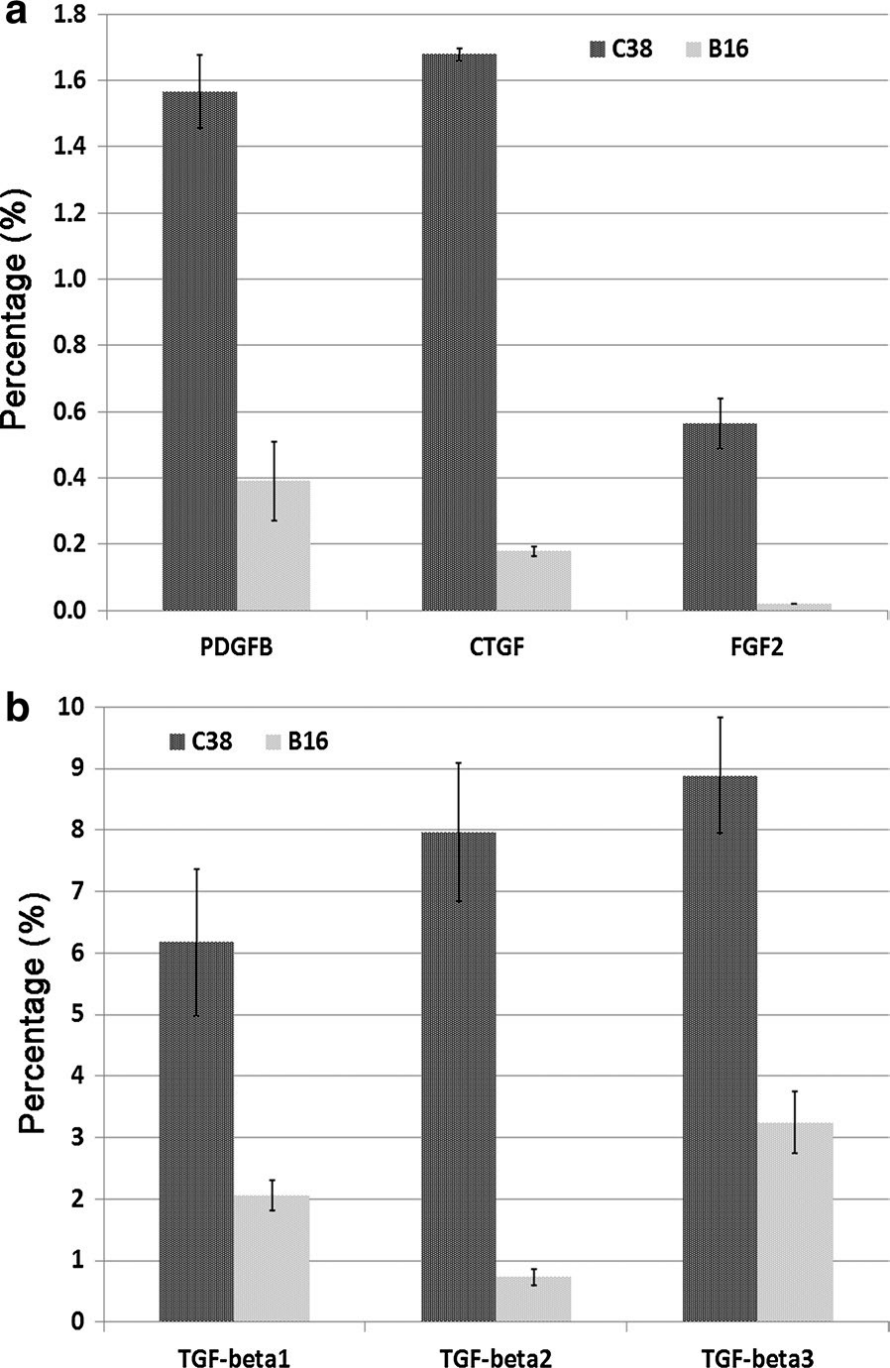
qRT-PCR analysis. qRT-PCR analysis of fibrogenic growth factors in B16 melanoma and C38 colon carcinoma cell lines. The relative expression levels (%) of Pdgfb, Ctgf, Fgf2 (**a**) and Tgf-β (**b**) were determined by comparing their expression levels to the expression level of GAPDH; the error bars display SD

In our earlier work, we analyzed in detail the vascularization of primary (intracutaneous) and metastatic (lung) B16 melanoma and found no accumulation of collagen at the tumor periphery or around the incorporated vessels (i.e., we did not observe intratumoral tissue columns in B16 tumors) [12, 14, 15]. Therefore, we used the B16 cell line as a control to compare the expression levels of fibrogenic factors with those of the column-inducing C38 tumor line. We found that the relative expression levels of Pdgfb, Fgf2, Ctgf (Fig. 5a) and Tgf-β (Fig. 5b) mRNAs were significantly higher in C38 cells.

## Discussion

In the present study, we analyzed the vascular and connective tissue structures of C38 colon carcinoma growing in different host tissues. The cecal wall represents the primary tumor site, whereas the skin, liver, lung, and brain tissues can be considered metastatic locations.

Tumor structure is dependent on the availability of tumor-derived fibrogenic factors and host tissue fibroblasts. Subcutaneous, cecal, hepatic, and pulmonary fibroblasts were peritumorally transformed into collagen-producing myofibroblasts. The evolution of tumorous connective tissue in these organs culminated in the appearance of intratumoral connective tissue columns with central vessels. The structure of these columns was identical in all of the aforementioned locations, resulting in a similar histological appearance of the tumors independent of the host organ. Therefore, it can be concluded that the tumor cells activate the peritumoral host tissue fibroblasts, which then participate in the formation of connective tissue columns. The activation of fibroblasts may be stimulated by the synthesis and secretion of tumor cell-derived “fibrogenic” factors. This concept, although requiring further studies, is supported by our observation that column-inducing C38 cells express higher levels of fibrogenic factors (e.g., Tgf-betas, Fgf2, Pdgfb, and Ctgf) [16–18] than B16 cells, which do not form tissue column-containing tumors in any locations [12, 14, 15]. In further support of our aforementioned findings, we observed that no intratumoral connective tissue columns developed in the brain (which lacks fibroblasts); instead, the capillaries of C38 cerebral metastasis were surrounded exclusively by tumor cells. Although previous studies suggest that pericytes can play an active role in connective tissue production [19–21], according to our results, mouse brain capillary pericytes did not produce connective tissue in the experimental conditions we used. It is also important to mention that intratumoral tissue columns were detected in cerebral C38 tumors when the metastatic tissue reached the meninges, where fibroblasts are located. In summary, the presence of the appropriate cell type (i.e., fibroblasts) in the tumor microenvironment and the synthesis of fibrogenic factors by the tumor cells can result in the same histological appearance of the tumor, regardless of the origin of the host tissue.

The early invaginations inevitably produced at the surface of the growing tumor spheres generally contain numerous incorporated vessels. Deeper in the tumor, the invaginations are “processed” (the tumor tissue pinches off connective tissue pieces, including vessels) down to the single-vessel stage (i.e., the connective tissue column produced contains only a single vessel). Although the size of these columns was fairly uniform in the different organs, we found significantly larger tissue columns in the skin compared with other organs. We cannot provide a reasonable explanation for this difference, as one would expect the diameter of the columns to be determined by the diffusion of oxygen and nutrients. However, tissue columns with the largest diameter can provide sufficient blood supply for several rows of tumor cells around the columns. It is also worth noting that the generation of intratumoral columns with such a regular structure is a unique characteristic of C38 tumors. Nevertheless, other tumor types also incorporate and process connective tissue (including the blood vessels) into rather irregular networks that are still able to provide tumors with oxygen and nutrients (unpublished data).

We found that the amount of connective tissue present in the columns and the integrity and thickness of the BM deposited by the tumor increased towards the tumor center, suggesting that the structures in the center of the tumor are older than those at the periphery. This finding is in line with the notion that “for tumor survival the edge is the future and the center is history” [22] and, moreover, provides additional proof that vascularization occurs by vessel incorporation, not vessel ingrowth [11–13, 23–31].

In conclusion, in the current study, we examined the microanatomy and the vascularization process of C38 colon carcinoma in five different organs using confocal and electron microscopy. Our results suggest that the vascular and connective tissue structures of tumors are determined by both the primary tumor type (i.e., the production of tumor-derived fibrogenic factors) and the host tissue microenvironment (i.e., the availability of suitable connective tissue elements).
